# Exploratory structural assessment in craniocervical dystonia: Global and differential analyses

**DOI:** 10.1371/journal.pone.0182735

**Published:** 2017-08-22

**Authors:** Larissa Vilany, Thiago J. R. de Rezende, Luiza G. Piovesana, Lidiane S. Campos, Paula C. de Azevedo, Fabio R. Torres, Marcondes C. França, Augusto C. Amato-Filho, Iscia Lopes-Cendes, Fernando Cendes, Anelyssa D’Abreu

**Affiliations:** 1 Neuroimaging Laboratory, School of Medical Sciences, State University of Campinas, Campinas, Brazil; 2 Chronology and Cosmic Rays Department, State University of Campinas, Campinas, SP, Brazil; 3 Neurology Department, School of Medical Sciences, State University of Campinas, Campinas, Brazil; 4 Medical Genetics Department, School of Medical Sciences, State University of Campinas, Campinas, Brazil; 5 Radiology Department—School of Medical Sciences, State University of Campinas, Campinas, Brazil; Institut du cerveau et de la moelle epiniere, FRANCE

## Abstract

**Introduction:**

Our goal was to investigate the cortical thickness and subcortical volume in subjects with craniocervical dystonia and its subgroups.

**Methods:**

We studied 49 subjects, 17 with cervical dystonia, 18 with blepharospasm or oromandibular dystonia, and 79 healthy controls. We performed a whole group analysis, followed by a subgroup analysis. We used Freesurfer software to measure cortical thickness, subcortical volume and to perform a primary exploratory analysis in the craniocervical dystonia group, complemented by a region of interest analysis. We also performed a secondary analysis, with data generated from Freesurfer for subgroups, corrected by false discovery rate. We then performed an exploratory generalized linear model with significant areas for the previous steps using clinical features as independent variables.

**Results:**

The primary exploratory analysis demonstrated atrophy in visual processing regions in craniocervical dystonia. The secondary analysis demonstrated atrophy in motor, sensory, and visual regions in blepharospasm or oromandibular dystonia, as well as in limbic regions in cervical dystonia. Cervical dystonia patients also had greater cortical thickness than blepharospasm or oromandibular dystonia patients in frontal pole and medial orbitofrontal regions. Finally, we observed an association between precuneus, age of onset of dystonia and age at the MRI exam, in craniocervical dystonia; between motor and limbic regions and age at the exam, clinical score and time on botulinum toxin in cervical dystonia and sensory regions and age of onset and time on botulinum toxin in blepharospasm or oromandibular dystonia.

**Conclusions:**

We detected involvement of visual processing regions in craniocervical dystonia, and a pattern of involvement in cervical dystonia and blepharospasm or oromandibular dystonia, including motor, sensory and limbic areas. We also showed an association of cortical thickness atrophy and younger onset age, older age at the MRI exam, higher clinical score and an uncertain association with longer time on botulinum toxin.

## Introduction

Dystonia is characterized by sustained or intermittent muscle contractions leading to abnormal movements, postures, or both. The movements are frequently initiated or worsened by voluntary action. They are typically patterned, twisting, and may be associated with tremor [1]. Craniocervical dystonia (CCD) comprises cervical dystonia (CD), blepharospasm (BSP), oromandibular (ORO), lingual and laryngeal (Lar) dystonia, isolated or associated [2]. Primarily thought of as a mainly basal ganglia circuits disorder, recent studies suggest the involvement of additional brain circuits and regions, in a wider network concept for dystonia [3, 4]. The physiopathology of dystonia is unclear. Despite some clinical particularities in different subtypes of CCD [2], some common features in dystonia’s cortical alterations [5] and genetics [6] have suggested a common physiopathology for its clinical subtypes.

Previous studies using voxel based morphometry (VBM) (see Table 1), have reported inconsistent patterns of morphologic changes. [5, 7–15] For instance, while older studies demonstrated increased [5, 8–11] or decreased [5, 7] grey matter (GM) in the basal ganglia, more recent ones [12–15] found no alterations in these regions. Previously we assessed CCD patients using VBM [14, 16], showing GM increase in the anterior cerebellum and brainstem (pons), along with GM decrease in the posterior lobe of cerebellum [16]. A whole brain analysis confirmed atrophy in the supplementary motor area [8, 11], premotor cortex [7, 8], precentral gyrus [7, 11, 12, 15], inferior parietal gyrus [9], superior temporal gyrus [5, 12], visual cortex [8], anterior cingulate [15], precuneus, middle temporal gyrus [11] and cerebellum [14]. We showed neither atrophy nor increase in GM in the basal ganglia [14].

**Table 1:** Literature review on previous study findings using voxel-based morphometry analysis in CCD.

First Authors (year)	DYTs^a^	p-value	Decreased GM^b^	Increased GM
Draganski (2003) [8]	CD^c^	<0.05 corrected	R^d^ caudal SMA^e^, R visual cortex, R dlPFC^f^	R GPi^g^, bilateral motor cortex, cerebellar flocculus
Etgen (2006) [9]	BSP^h^	<0.001 uncorrected	L^i^ inferior parietal lobule	Bilateral putamen
Egger (2007) [10]	CD	<0.05 corrected	-	Bilateral orbitofrontal cortex, R GPi, medial frontal gyrus, L SMA and cingulate gyrus
Obermann (2007) [5]	BSP	<0.05 corrected	Bilateral thalamus, putamen	Bilateral caudate head, cerebellum
	CD	<0.05 corrected	Bilateral putamen, superior temporal lobule	Bilateral caudate head, thalamus, L posterior cerebellar lobe and superior temporal lobule
Suzuki (2011) [13]	BSP	<0.05 corrected	-	Bilateral sensorimotor cortices, L cingulate
Martino (2011)* [12]	BSP	<0.05 corrected	L superior temporal gyrus	R middle frontal gyrus
		<0.001 uncorrected	R Uncus, R subgyral region, L postcentral and precentral gyrus	Bilateral superior frontal gyrus, R middle frontal gyrus, L anterior cingulated
Pantano (2011) [7]	CD	<0.05 corrected	Bilateral premotor and primary sensorimotor cortices, L caudate head, putamen	-
Prell (2013) [11]	CD	<0.001 uncorrected	L precentral, SMA, medial temporal gyrus and R somatosensory association cortex	L GPi, frontal eye field, R claustrum, putamen and bilateral medial surface of occipital lobe
Horovitz (2012)* [15]	BSP	<0.01 uncorrected	R orbitofrontal, L precentral, inferior frontal, R occipital cortex and anterior cingulate gyrus.	L middle temporal, R postcentral, bilateral precuneus
Piccinin (2015)* [17]	CCD^j^	<0.001 uncorrected	Cerebellar vermis, bilateral superior frontal gyrus, precuneus, anterior cingulated, paracingulate, insular cortex, lingual gyrus and calcarine fissure; L SMA, inferior frontal, inferior parietal, temporal pole, supramarginal, rolandic operculum, hippocampus, parahippocampal, middle occipital, cerebellar lobules IV/V, superior and middle temporal gyri; R middle cingulate and precentral	-

^a^DYTs: dystonia subtypes

^b^GM: grey matter

^c^CD: cervical dystonia

^d^R: right

^e^SMA: supplementary motor area

^f^dlPFC: dorso lateral prefrontal cortex

^g^GPi: globus pallidus internum

^h^BSP: blepharospasm

^i^L: left

^j^CCD: craniocervical dystonia

*images acquired at 3T MRI machines; otherwise images were acquired in 1.5 MRI machines

Freesurfer (FS) is software that performs an automatic segmentation of the cerebral cortex and subcortical structures, generating measures such as area, volume and cortical thickness. FS has good validation and accuracy in neurological disorders [18, 19] and has better sensitivity than VBM, when applied to cortical thickness [20].

Our primary goal was to investigate the differences in cortical thickness and subcortical volume between patients with CCD and controls, and to investigate possible relationships between these regions and clinical variables, such as age of onset, clinical score on the Fahn-Marsden scale (FMS) and time of treatment with botulinum toxin (BoNT). Our secondary goal was to determine if there were different patterns of cerebral involvement within subgroups of our sample, cervical dystonia isolated (CD), blepharospasm or oromandibular dystonia, isolated or combined (B&O). Both goals were successfully accomplished.

## Methods

### Subjects

This was a cross-sectional study conducted at the Movement Disorders and the Dystonia Outpatient Clinics and the Neuroimaging Laboratory at the University of Campinas (UNICAMP). The Ethics Committee of the School of Medical Sciences of the University of Campinas approved the study under the number 161/2010 and all subjects signed an informed consent prior to study entry, in accordance with the Declaration of Helsinki.

We recruited subjects consecutively at the Movement Disorders and the Dystonia Outpatient Clinics. The inclusion criteria were: a clinical diagnosis of dystonia, with or without tremor, and an otherwise normal neurological examination; no history of use of medications known to cause dystonia; a magnetic resonance image (MRI) without significant abnormalities; and the ability to provide informed consent. The exclusion criteria were history of alcoholism, abnormal neurological exam, other neurological disorder, secondary dystonia. We thoroughly reviewed the clinical and family histories, performed a complete and detailed neurological examination, including the FMS. Two Movement Disorders Neurologists examined all study participants (LGP and AD).

Our sample consisted of 51 patients with CCD and 82 healthy controls gathered from the same population base. Healthy controls had no history of neurological or psychiatric diseases, a normal clinical and neurological examination and normal neuroimaging studies. We excluded images of 2 patients and 3 controls due to artifacts. From the 49 resultant CCD patients, 17 had CD and 18 B&O. Twenty-five individuals had focal dystonia (17 CD, 6 BSP, 2 ORO) and 24 had segmental dystonia (1 BSP/CD, 10 BSP/ORO, 2 BSP/ORO/Lar, 8 BSP/CD/ORO, 1 CD/Lar, 1 ORO/CD, 1 ORO/Lar). All tested negative for DYT1 and DYT6, the most common mutations in genetic-determined cases of dystonia [21].

Mean age of patients and control groups were 60.39±13.99 and 57.52±9.85 years, respectively (p-value = 0.2129). Our gender distribution included 34 women (69.38%), and 15 men (30.61%) in the CCD group, and 44 women (55.7%), and 35 men (44.3%) in the control group (X^2^ = 2.3816, p = 0.123). Mean age of onset of dystonia syndrome was 51± 13.78 years and mean FMS score was 5.94±3.28. Median time of BoNT treatment was of 2.5 years (minimum of 0, maximum of 17), last injection at most 30 days prior to MRI scanning. All the subjects who were evaluated in our previous studies [14, 16] were included in this one. Table 2 details demographics of the studied population.

**Table 2:** Subjects demographics.

	Subjects Demographics			
	CCD (n = 49)	CD (n = 17)	B&O (n = 18)	Controls (n = 79)
**Age (y)**	60.39 ± 13.99*	52.06 ± 13.11**	63.83 ± 11.83***	57.52 ± 9.85
**Gender (male)**	15 (30.61%)	7 (41.18%)	4 (22.22%)	35 (44.30%)
**Age of onset (y)**	51 ± 13.78	41 ± 13.39	56 ± 10.86	
**Score MF**	5.94±3.28	3.44±0.25	5.61 ± 2.32	
**Mean Time on BoTN (y)**	2.5 (0–17)	7 (0–17)	1(0–17)	

Difference between mean ages of patients groups and controls

*(p = 0.2129) (ttest)

**(p = 0.1205)

***(p = 0.0468). y = years

### MRI acquisition

We acquired images in a 3T-Achieva-Intera magnetic resonance scanner–Philips, in a T1 volumetric sequence, according to the following parameters: isotropic voxels of 1mm^3^, acquired in sagittal plane (1mm of thickness; flip angle: 8°; repetition time: 7.1ms; echo time: 3.2ms; matrix: 240x240; and field-of-view: 240x240 mm).

### FS analyses

We used FreeSurfer software v.5.3 to obtain measurements according to the protocol suggested by Fischl & Dale [22]. A correction for magnetic field inhomogeneity was performed. Images were aligned to the Talairach and Tournoux atlas [23] and skull-stripped. Subsequently, voxels were labeled as grey matter (GM), white matter and cerebral spinal fluid. Triangle meshes were then used to establish a pial surface and a white surface, which is the interface between grey and white matter.[22] The shortest difference between these surfaces, vertex by vertex, resulted in the measure of cortical thickness. For smoothing of surfaces, a Gaussian filter with 10 mm full width at half-maximum was used for all analyses. Additionally, the calculations of estimated total intracranial volume [24] and subcortical structures volume were performed [25]. We used an automatic General Linear Model (GLM) in Freesurfer’s application QDEC (Query, Design, Estimate, Contrast) to determine regional variations of cortical thickness between patients and controls. Since this was an exploratory analysis, we used an uncorrected p-value to identify significant vertex-wise group clusters in CCD patients.

#### Statistics

We performed two analyses: a primary analysis–total CCD patients versus controls, and a secondary analysis–CD and B&O versus controls.

Besides vertex-wise comparisons, FreeSurfer generates cortical thickness and subcortical volume measurements for parcellation [25, 26] based on an anatomical atlas [27]. Our primary analysis, in CCD, included cortical thickness in a vertex-wise analysis and was already previously described. We also performed a region of interest (ROI) analysis with subcortical region volumes for their presumed role in movement disorders, that were not contemplated in our previous method, such as putamen, caudate, thalamus, pallidum and cerebellum for all groups. We applied the FS—Desikan atlas [27] for the selected ROIs, then executed a GLM with age, gender and estimated total intracranial volume as independent variables to evaluate for significant differences in subcortical volumes between patients and controls for each region in CCD. We applied the false discovery rate method (FDR) for correction, p<0.05. For this analysis, we used Stata v 13.1.

Our secondary analysis also consisted in a ROI analysis, for cortical thickness and subcortical volumes, between CD, B&O and control groups. We executed a Kruskal-Wallis test, with age, gender and estimated total intracranial volume as independent variables to evaluate for significant differences in cortical thickness and subcortical volume between patient subgroups among themselves and controls for each region, according to FS-Desikan atlas [27], also corrected by FDR. Subsequently, we performed a Mann-Whitney test corrected by Bonferroni. This analysis was performed in Partek v 6.6.

#### Clinical-anatomical correlations

We performed a GLM in which the dependent variable was the region statistically different between patients and controls at the previous step. Independent variables were age at the MRI exam, age of onset of dystonia, FMS and time on BoNT. Two subjects of our original 49 patients sample were excluded due to missing clinical data. This analysis included 47 subjects with CCD, 15 with CD and 18 with B&O. We used Stata v13.1 for this analysis. We present an exploratory and a FDR corrected analysis.

## Results

**Fig 1:**
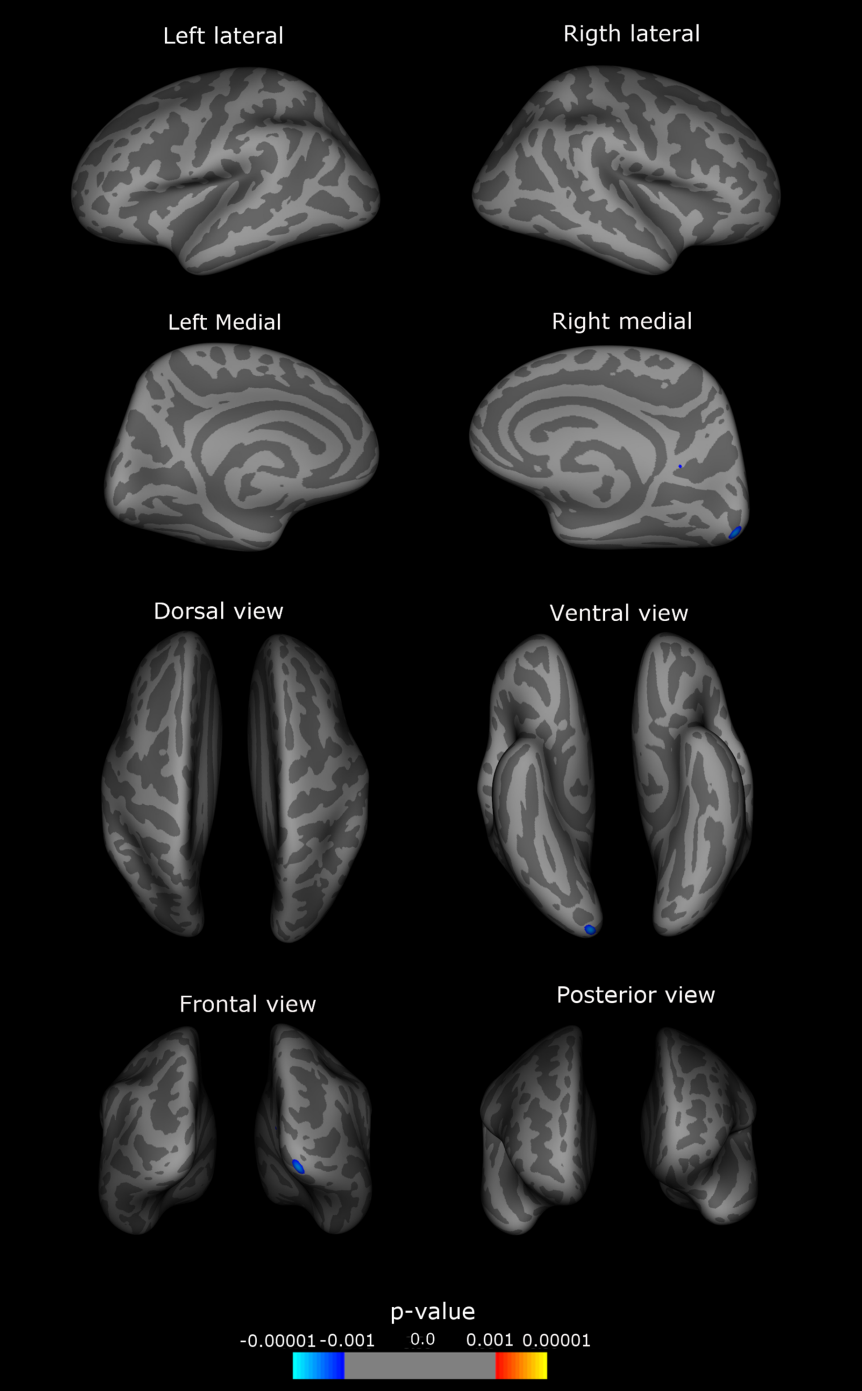
Exploratory analysis. Right and left hemispheres, lateral, medial, frontal, posterior, superior and inferior views. Atrophic regions are marked in blue, regions with increased cortical thickness in red, colorbar indicates p-value. In light grey shade, sulci, in dark grey shade, gyri. Significant different regions from the control group: CCD: right precuneus and lateral occipital.

Exploratory primary vertex-wise analyses showed decreased cortical thickness (cortical atrophy) in the right precuneus and lateral occipital gyrus in CCD (Fig 1) (Table 3).

**Table 3:** Primary exploratory analysis.

Cortical Area	Cluster size (mm^2^)	TalX	TalY	TalZ	Patients±Sd^a^ mean (mm)	Controls±Sd mean (mm)	p-value
CCD							
Right Lateral occipital	141.29	16.7	-98.0	-10.4	1.96±0.16	1.99 ±0.14	0.000
Right Precuneus	4.91	9.8	-56.5	15.5	2.11±0.16	2.19 ±0.12	0.001

Table 3 –Primary exploratory analysis, CCD group—atrophic cortical areas, clusters data, mean cortical thickness and statistical significance.

^a^Sd: standard deviation TalX, TalY, TalZ: spatial coordinates of the clusters, axis x, y, z (height, width, depth

**Fig 2:**
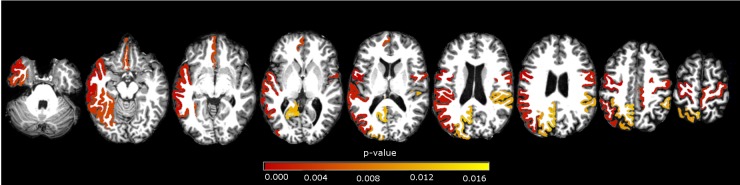
ROI subgroups analysis. Axial view of significant areas in the comparison of CD versus controls. Areas described in Table 4. p-values in red scale colorbar, α = 0.017 (Bonferroni correction).

**Fig 3:**
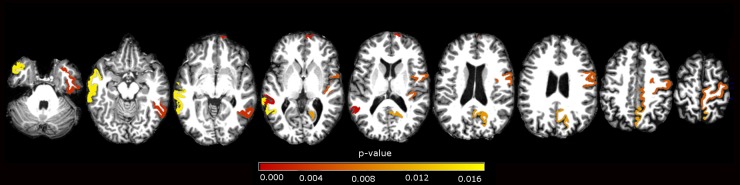
ROI subgroups analysis. Axial view of significant areas in the comparison of B&O versus controls. Areas described in Table 4. p-values in red scale colorbar, α = 0.017 (Bonferroni correction).

**Fig 4:**
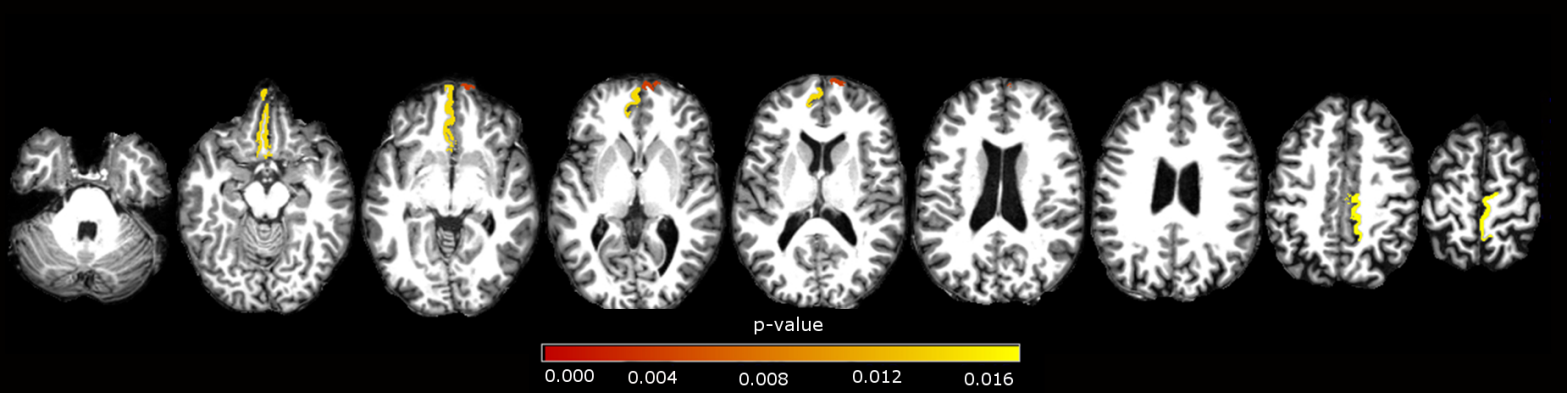
ROI subgroups analysis. Axial view of significant areas in the comparison of CD versus B&O. Areas described in Table 4. p-values in red scale colorbar, α = 0.017 (Bonferroni correction).

At our secondary ROIs analysis, we showed atrophy in left fusiform, inferior parietal, inferior temporal, isthmus of cingulate, medial orbitofrontal, middle temporal, precentral, precuneus, superior parietal, superior temporal, supramarginal gyri and right paracentral, precentral, supramarginal gyri in the comparison of CD and controls. We demonstrated cortical atrophy in left posterior portion of the superior temporal sulcus, middle temporal gyrus, and right inferior temporal, paracentral, precuneus, frontal pole and transverse temporal region, when comparing B&O to controls. In the CD versus B&O analysis, we found higher cortical thickness in the CD group in left medial orbitofrontal and right frontal pole (see Table 4 and Figs 2–4).

There were no significant differences in subcortical structure volumes between any of the groups.

**Table 4:** ROI analysis.

**Cortical Area**	**p-value**	**Patients ± Sd**^**a**^ **mean (mm)**	**Controls ± Sd mean (mm)**
**CD x Ctl**^**b**^			
Left Fusiform	0.004	2,56±0,21	2,64±0,16
Left Inferior parietal	0.001	2,15±0,15	2,20±0,12
Left Inferior temporal	0.003	2,59±0,19	2,63±0,16
Left Isthmus cingulate	0.011	2,41±0,28	2,46±0,22
Left Medial orbitofrontal	0.004	2,37±0,17	2,34±0,15
Left Middle temporal	0.000	2,55±0,20	2,66±0,16
Left Precentral	0.000	2,30±0,18	2,36±0,13
Left Precuneus	0.012	2,19±0,14	2,24±0,13
Left Superior parietal	0.010	1,94±0,16	1,96±0,11
Left Superior temporal	0.001	2,55±0,24	2,62±0,16
Left Supramarginal	0.001	2,28±0,14	2,32±0,11
Right Paracentral	0.002	2,14±0,15	2,23±0,13
Right Precentral	0.001	2,26±0,18	2,35±0,13
Right Supramarginal	0.011	2,26±0,17	2,33±0,13
**B&O x Ctl**	**p-value**	**Patients ± Sd mean (mm)**	**Controls ± Sd mean (mm)**
Left Bankssts^**c**^	0.000	2,20±0,13	2,32±0,15
Left Middle temporal	0.015	2,52±0,12	2,66±0,16
Right Inferior temporal	0.003	2,59±0,15	2,71±0,15
Right Paracentral	0.006	2,13±0,15	2,23±0,13
Right Precentral	0.005	2,25±0,11	2,35±0,13
Right Precuneus	0.010	2,11±0,13	2,19±0,12
Right Frontal pole	0.001	2,58±0,20	2,61±0,21
Right Transverse temporal	0.005	2,08±0,20	2,17±0,22
**CD x B&O**	**p-value**	**CD ± Sd mean (mm)**	**B&O ± Sd mean (mm)**
Left Medial orbitofrontal	0.014	2,37±0,17	2,20±0,14
Right Frontal pole	0.004	2,82±0,34	2,58±0,20

Table 4—ROI analyses, for CD and B&O groups. Mean cortical thickness, standard deviation, and p-values, indicating areas with decreased cortical thickness. No significant results were detected in subcortical structures.

^a^Sd: standard deviation

^b^ Ctl: controls

^c^bankssts: posterior portion of the superior temporal sulcus

In CCD we observed that the greater the age at symptom onset, the higher the cortical thickness in the right precuneus, and the older the subject at MRI exam, the smaller the cortical thickness in the same area. In CD, we found that patients with higher scores in the FMS and longer time receiving toxin treatments had reduced cortical thickness in the right precentral gyrus and the older the patient at the MRI exam, the greater the atrophy in left isthmus of the cingulate gyrus. Additionally, in B&O, older subjects at symptom onset had greater cortical thickness in the right inferior temporal gyrus and right precuneus. The longer time on BoNT, the higher the cortical thickness in the left middle temporal and right inferior temporal gyri. And finally, the older the patient at the time of MRI exam, the more severe the atrophy in right inferior temporal gyrus. When comparing CD and B&O, we observed that the higher the score on FMS, the smaller the cortical thickness in CD group in left medial orbitofrontal area compared to B&O. None of these associations survived FDR correction. (Table 5)

**Table 5:** Clinical analysis.

**CCD x Ctl**^a^					
**Cortical Area**	**Clinical Variable**	**p-value**	**FDR**^b^	**Coefficient**	**95%CI**^c^
Right precuneus	Age at MRI^d^	0.009	0.072	-0.010	-0.0178, -0.0025
Right precuneus	Age of onset	0.023	0.092	0.009	0.0012, 0.0169
**CD x Ctl**					
**Cortical Area**	**Clinical Variable**	**p-value**	**FDR**	**Coefficient**	**95%CI**
Left Isthmus cingulate	Age at MRI	0.015	0.390	-0.032	-0.0574, -0.0062
Right Precentral	Score FMS^e^	0.038	0.439	-0.310	-0.6026, -0.0173
Right Precentral	Time BoNT^f^	0.024	0.416	-0.020	-0.0387, -0.0027
**B&O x Ctl**					
**Cortical Area**	**Clinical Variable**	**p-value**	**FDR**	**Coefficient**	**95%CI**
Left Middle temporal	Time BoNT	0.039	0.405	0.015	0.0008, 0.0297
Right Inferior temporal	Age at MRI	0.004	0.208	-0.015	-0.0252, -0.0048
Right Inferior temporal	Age of onset	0.000	0.000	0.019	0.0083, 0.0296
Right Inferior temporal	Time BoNT	0.025	0.371	0.016	0.0021, 0.0313
Right Precuneus	Age of onset	0.031	0.403	0.012	0.0011, 0.0234
**CD x B&O**					
**Cortical Area**	**Clinical Variable**	**p-value**	**FDR**	**Coefficient**	**95%CI**
Left Medial orbitofrontal	Score FMS	0.045	0.425	-0.033	-0.0648, -0.0007

Table 5 –Clinical anlysis. Cortical areas and clinical variable related, exploratory data and correction by FDR.

^a^Ctl: control group

^b^MRI: magnetic resonance image

^c^FDR: false discovery rate

^d^CI: confidence interval

^e^FMS: Fanh-Marsden scale

^f^BoNT: botulinum toxin

## Discussion

This is the first study we are aware of to apply FS to CCD and to also investigate differences in structural involvement in CD and B&O. We used this method because neuroimaging and histological studies have validated FS measurements [18, 22], and FS has shown superior sensitivity in assessing cortical thickness when compared to VBM [20]. We found cortical atrophy in visual processing regions in CCD. We found atrophy in visual, sensory and motor areas in B&O and CD, the latter also showing cortical atrophy in a limbic area. We demonstrated a decrease in cortical thickness related to ageing, a positive association between earlier age at symptom onset and the severity of cortical atrophy, and a negative association between FMS scores and cortical thickness. We also found a negative association between time on BoNT and motor cortical thickness in CD and a positive association with temporal areas in B&O.

We confirmed atrophy in the precentral gyrus [7, 8, 11, 13–15], the superior temporal gyrus [5, 12, 14], the SMA [8, 10, 11, 14], the supramarginal gyrus [11, 14], the middle temporal gyrus [11, 14, 15], the inferior parietal gyrus [9, 11, 14], the precuneus [11, 14, 15], as demonstrated in previous studies. We found no changes in GM in the basal ganglia. [12–15]

Dystonia probably results from a loss of inhibition and increased plasticity, leading to abnormalities in sensorimotor integration and mal-adaptive plasticity [4, 28], affecting the basal ganglia-thalamo-cortical circuit, and the cerebello-thalamo-cortical circuit [3]. Defects in sensorimotor integration and alterations in neuropsychiatric features and sleep [29] have also been reported in dystonia patients.

The precentral gyrus, SMA, prefrontal and premotor cortex, basal ganglia, thalamus and cerebellum take part in the motor planning, control and execution of movement.[30] Patients with dystonia have abnormalities in multiple parts of these circuits [7, 8, 10, 11, 13–15], and increased connectivity in the executive control network [31].

There are also structural [5, 7, 11–13] and functional [29] abnormalities in multiple sensory processing areas in dystonic patients. Sensory function is an important component in motor execution. The sensorimotor network includes sensorimotor cortex, secondary somatosensory cortex, postcentral gyrus, frontal and parietal regions, basal ganglia and cerebellum [32]. Possible clinical correlates of sensory involvement are the sensory tricks and the presence of symptoms such as pain in CD, irritation or dry eyes in BSP [33]. Studies in focal dystonia demonstrated abnormalities in cortical representation, increases in spatial and temporal discrimination thresholds in affected and unaffected parts, abnormal perception of movement, altered cognitive representation of movement, impaired sensorimotor integration, abnormal connectivity between sensory and frontal cortices, especially in the premotor and parietal regions [32]. Although some widespread dysfunction in brain networks (in cortical and subcortical regions) was demonstrated, the results are still contradictory, in regarding to increases or decreases in connectivity between those regions.[32]

The primary visual network includes the occipital cortex, prefrontal cortex, premotor, superior parietal lobule, middle and inferior temporal gyri. It is integrally involved in visual perception, the processing of spatial information regarding spatial cognition, and sensorimotor integration, for visual input related to movement planning and control.[31] Besides sensorimotor impairment, dystonia patients also have a reduced connectivity among visual areas and worse performance in visual perception and visuospatial tasks [31]. The cingulate cortex is part of the limbic circuit, which is related to psychiatric conditions, attention processing and emotions [34]. Depression, anxiety, pain, and sleep disorders have an increased prevalence in dystonia patients, particularly in CD [29].

We observed no differences in subcortical GM structures, such as the basal ganglia, thalamus and cerebellum. Recent studies [12–15] also failed to demonstrate structural basal ganglia involvement, while older ones reported conflicting results [5, 7–11]. The basal ganglia and the thalamus are probably affected functionally rather than structurally. FS has good accuracy and reproducibility in assessing cortical and subcortical structures [18, 19, 22], with good correlations between its cortical thickness and measures obtained manually by experienced anatomists [19, 22]. Some studies [3, 5, 14] have demonstrated structural involvement of specific cerebellum regions. FS cerebellum segmentation evaluates grey and white matter in the left and right hemispheres [25], with no account of subdivisions or the vermis.

Clinical-morphological associations have not been widely observed in dystonia [7, 12]. Some studies suggest that focal brain atrophy is worse in subjects with earlier disease onset, longer disease duration, higher disability, as measured by clinical scales scores and duration of BoNT treatment [9, 13, 14]. It is unclear if this association is related to treatment itself, since BoNT has been shown to alter neuromodulation [31], or whether it simply reflects a longer disease duration. Conversely, other results show symptom progression correlating with increased GM volume in some regions [11, 14]. Early onset age is usually associated with more severe cases of dystonia [1], possibly justifying therefore, greater cortical atrophy in such patients. Age and age of onset are not correlated in dystonia, only the latter being used for current classification purposes [1]. Cortical atrophy has also been demonstrated with normal ageing, as we observed in some regions, such as precuneus, isthmus of the cingulate and inferior temporal gyrus [35]. These results, however, were exploratory, possibly due to a discrete association between variables, since by the high number of comparisons, p-values did not sustain FDR correction.

We found similarities and differences in the pattern of atrophy between CD and B&O. Differences between CCD and subgroups (CD and B&O) would be expected, since the whole group also included subjects with other subtypes of dystonia or more complex presentations. CD demonstrated decreased GM, especially in the sensorimotor and visual processing areas, such as B&O. The common involvement of sensorimotor and visual areas in both subgroups reinforces the hypothesis of a common pathway in the development of dystonias [5, 6], while the differences in cerebral involvement observed may provide the physiopathological basis for the different clinical presentations. CD is the most common form of dystonia, usually presents around the fifth decade of life, and may be accompanied by pain and show periods of remission, usually transitory. BSP usually presents by the fifth to seventh decade of life, has sensory tricks and has an association with ocular symptoms, functional blinding and other neurological conditions (with a high level of limitation and comorbidities), commonly progressing to Meige’s syndrome (BSP associated with ORO). ORO may have a spontaneous start, be triggered by actions such as eating or speaking and is frequently associated with BSP.[2]

Limitations of this study were the small size and heterogeneity of our sample, as well as the small size of our subgroups. However, we included a group of subjects not yet investigated, such as those with ORO and segmental dystonias. We also did not take into consideration the laterality of the symptom presentation and dominance, especially in those with cervical dystonia. Yet, to address laterality in CCD is a difficult task, since most patients show a complex range of movements in CD, bilateral involvement in BSP and laryngeal dystonia, and a complex pattern of muscle involvement in ORO.

## Conclusion

FS demonstrated the involvement of areas involved in visual processing in CCD. There were similarities and differences in the pattern of involvement in CD and B&O, which may reinforce the hypothesis of a common physiopathology and also explain some of the clinical differences between them.
